# Supplementary figures and images for: Infected connections: Unraveling the impact of a bacterial symbiont on ant-aphid partnership
Source: PLoS One. 2025 Jun 23;20(6):e0326875. doi: 10.1371/journal.pone.0326875 (PMC12184899; doi:10.1371/journal.pone.0326875)

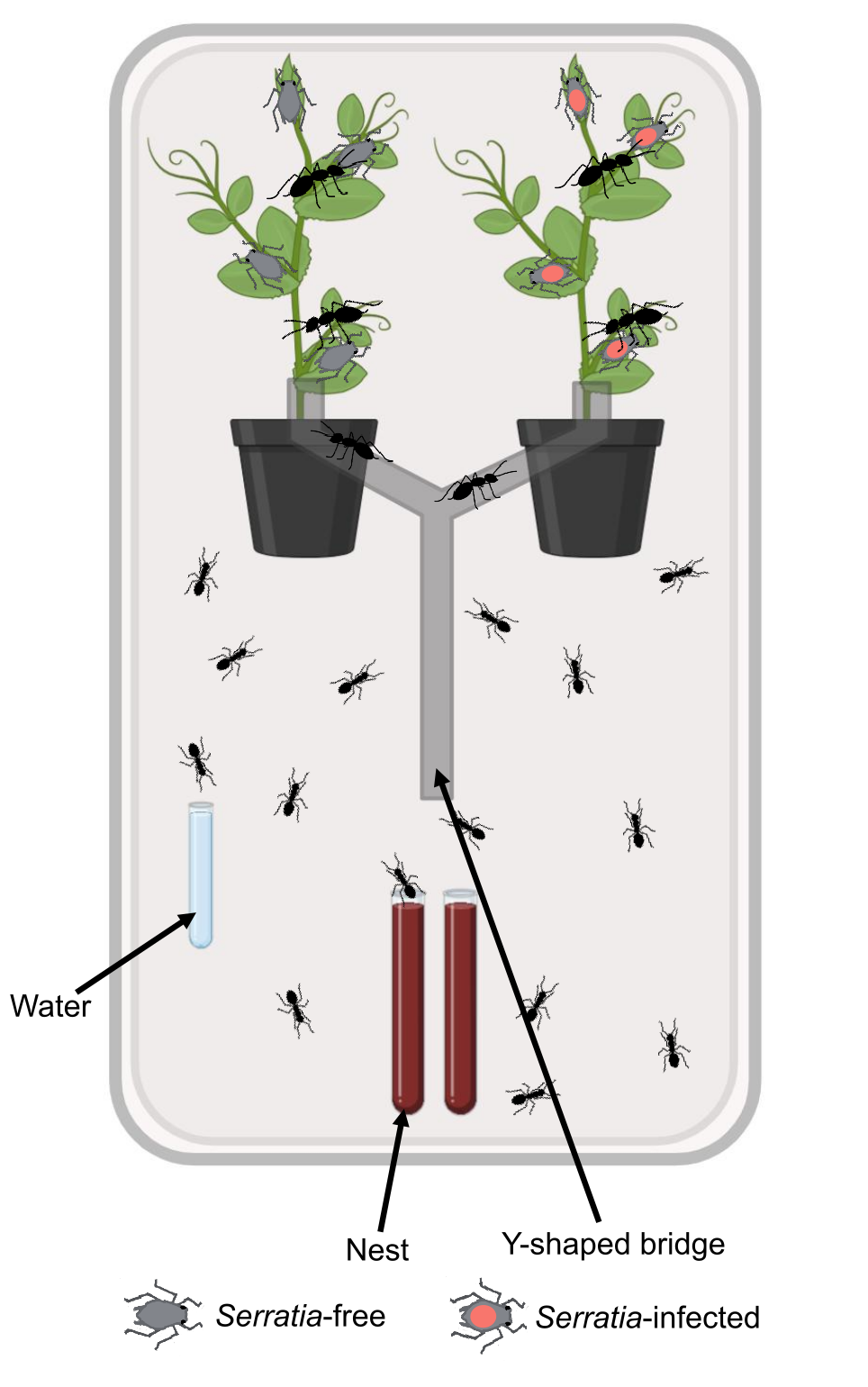

Supplement: S1 Fig — (TIF) [file pone.0326875.s001.tif]

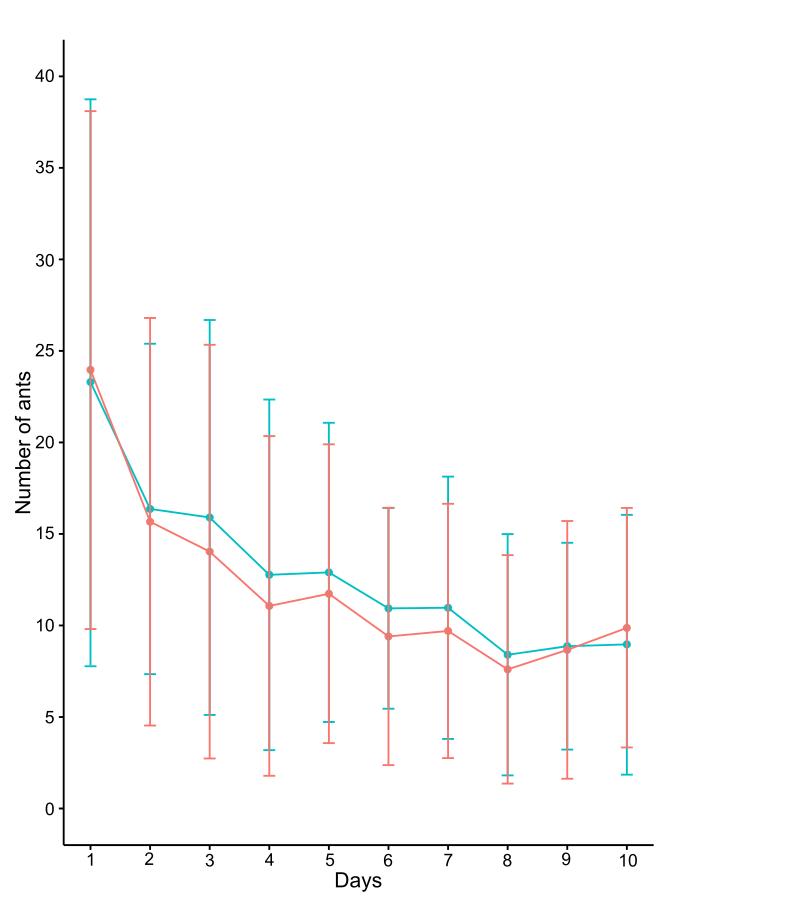

Supplement: S2 Fig — Average values are represented by points and were calculated over 10 replicates for each infection status, error bars represent standard deviation. (TIF) [file pone.0326875.s002.tif]

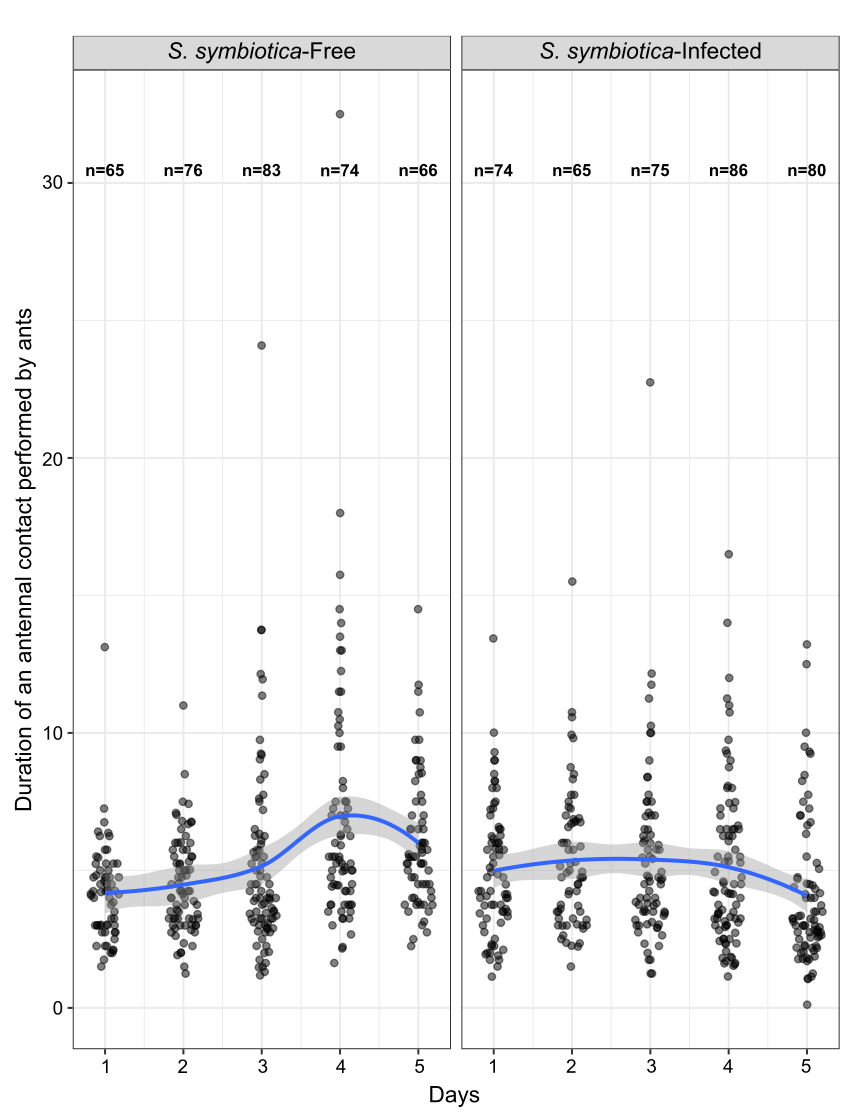

Supplement: S3 Fig — Each grey point represents a contact event, n = 364 on S. symbiotica-free aphids and n = 380 on S. symbiotica-infected aphids over 10 replicates for each infection status. The number of antennal contacts observed are indicated for each day. The blue trend line is modeled by “flexplot” function in R with the assumption of a Gaussian distribution and shows the general pattern of duration of antennal contact over time. (TIF) [file pone.0326875.s003.tif]

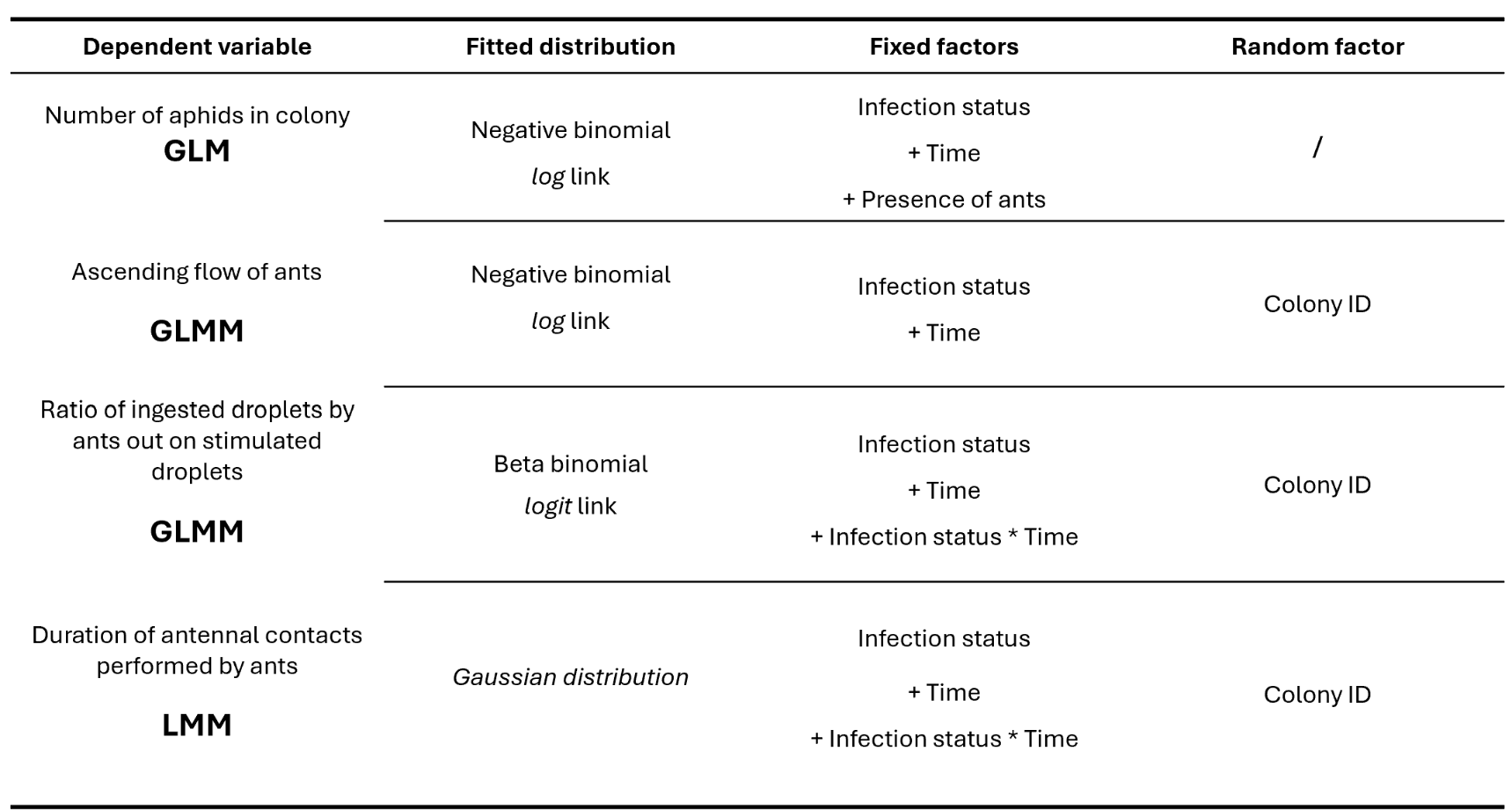
 S1 Table. Description of models used for the statistical analyses.

Supplement: S1 Table — (DOCX) [file pone.0326875.s004.docx]
