# Supplementary material for: Infected connections: Unraveling the impact of a bacterial symbiont on ant-aphid partnership
Source: PLoS One. 2025 Jun 23;20(6):e0326875. doi: 10.1371/journal.pone.0326875 (PMC12184899; doi:10.1371/journal.pone.0326875)
Supplement: S2 Table — Models were compared using maximum likelihood estimate of the model (log10L), Akaike’s Information Criterion corrected (AICc). Delta AIC and the degree of freedom (df) are also indicated. Models are ranked by increasing values of AICc. The arrow indicates the best model considered. (DOCX) [file pone.0326875.s005.docx]

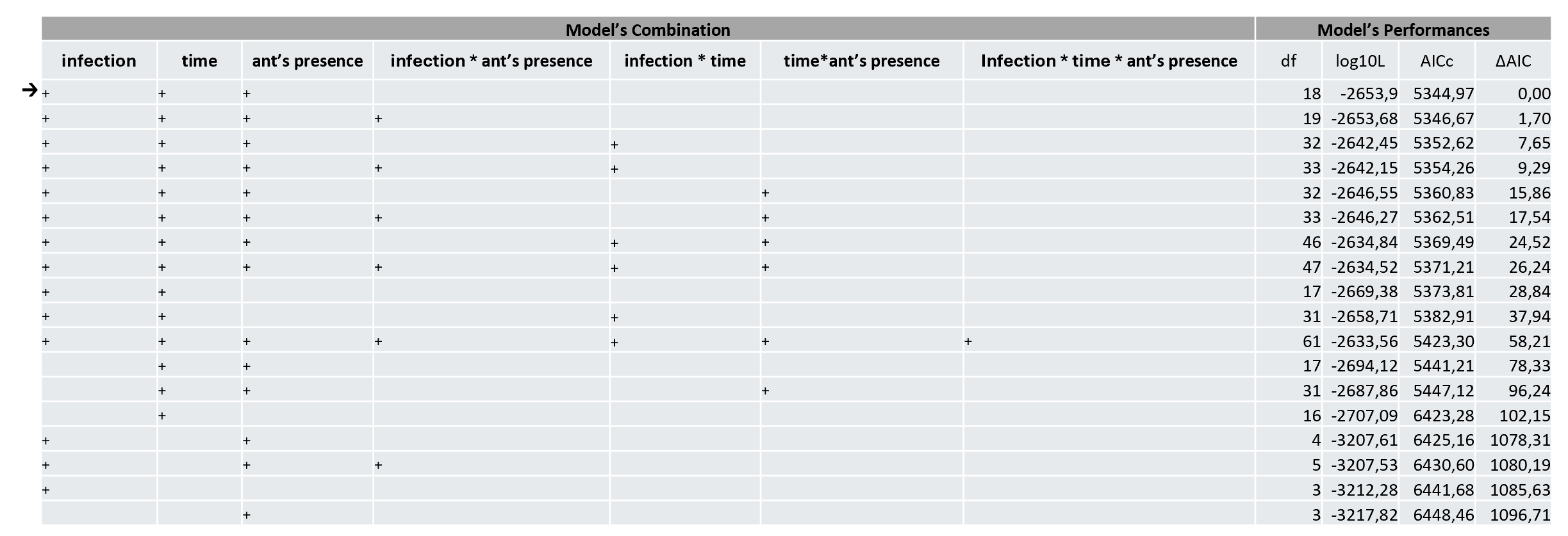
 **S2 Table. Model comparison for the growth dynamics of aphid populations**, considering the infection status, time and presence of ants as fixed factors as well as first and second order interaction effects. Models were compared using maximum likelihood estimate of the model (log10L), Akaike’s Information Criterion corrected (AICc). Delta AIC and the degree of freedom (df) are also indicated. Models are ranked by increasing values of AICc. The arrow indicates the best model considered.
