# Supplementary material for: Infected connections: Unraveling the impact of a bacterial symbiont on ant-aphid partnership
Source: PLoS One. 2025 Jun 23;20(6):e0326875. doi: 10.1371/journal.pone.0326875 (PMC12184899; doi:10.1371/journal.pone.0326875)
Supplement: S4 Table — Models were compared using maximum likelihood estimate of the model (log10L), Akaike’s Information Criterion corrected (AICc). Delta AIC and the degree of freedom (df) are also indicated. Models are ranked by increasing values of AICc. The arrow indicates the best model considered. (DOCX) [file pone.0326875.s007.docx]

**
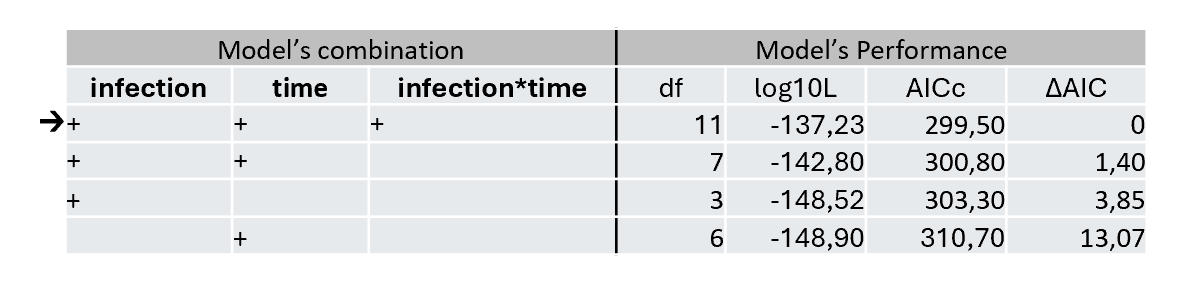
S4 Table**. **Model comparison for the proportion of consumed droplets by ants**, with the infection status and time as fixed factors as well as first order interaction effect, the random factor for all models is the ant colony. Models were compared using maximum likelihood estimate of the model (log10L), Akaike’s Information Criterion corrected (AICc). Delta AIC and the degree of freedom (df) are also indicated. Models are ranked by increasing values of AICc. The arrow indicates the best model considered.
