# Supplementary material for: Infected connections: Unraveling the impact of a bacterial symbiont on ant-aphid partnership
Source: PLoS One. 2025 Jun 23;20(6):e0326875. doi: 10.1371/journal.pone.0326875 (PMC12184899; doi:10.1371/journal.pone.0326875)
Supplement: S6 Table — As for the time effect, experimental days that shared a common letter were not significantly different when using Tukey’s post-hoc tests. (DOCX) [file pone.0326875.s009.docx]

**
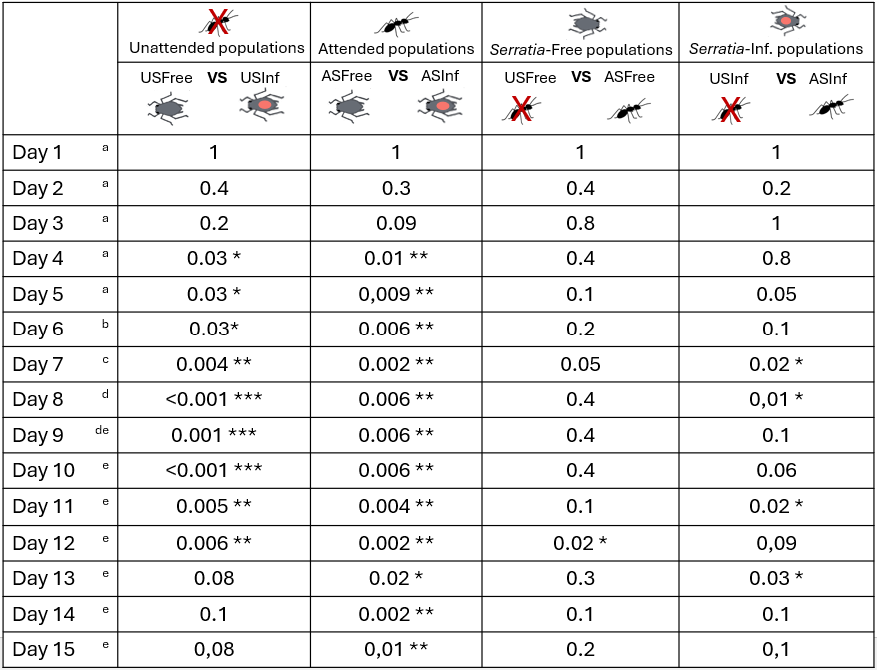
S6 Table. P-values of Wilcoxon signed rank tests with Bonferroni adjustment,** comparing the effects of aphid infection status per day and the effects of attendance by ants per day (*, p<0.05; **, p<0.01; ***, p<0.001). As for the time effect, experimental days that shared a common letter were not significantly different when using Tukey’s post-hoc tests.
