# Supplementary material for: Infected connections: Unraveling the impact of a bacterial symbiont on ant-aphid partnership
Source: PLoS One. 2025 Jun 23;20(6):e0326875. doi: 10.1371/journal.pone.0326875 (PMC12184899; doi:10.1371/journal.pone.0326875)
Supplement: S7 Table — N values are the numbers of observations per infection status of aphids and time for the proportion of droplets consumed by ants after having stimulated honeydew emission and for the duration of antennal contacts. Means and standard error values are provided,as well as p-values for fixed factors and interaction effect (*, p < 0.05; **,p < 0.01; ***, p < 0.001), the ant’s colony is used as random factor. Post-hoc multiple pairwise comparisons (PWC) were conducted and adjusted using Tukey’s method, p-values are indicated (*, p < 0.05; **, p < 0.01; ***, p < 0.001) and experimental days sharing a common letter are not significantly different. (DOCX) [file pone.0326875.s010.docx]

**S7 Table. Consumption of honeydew droplets by ants over the course of the experiment.**

N values are the numbers of observations per infection status of aphids and time for the proportion of droplets consumed by ants after having stimulated honeydew emission and for the duration of antennal contacts. Means and standard error values are provided, as well as p-values for fixed factors and interaction effect (*, p<0.05; ***, p<0.001), the ant’s colony is used as random factor. Post-hoc multiple pairwise comparisons (PWC) were conducted and adjusted using Tukey’s method, p-values are indicated (*, p<0.05; **, p<0.01; ***, p<0.001) and experimental days sharing a common letter are not significantly different.

|  | Proportion of ingested droplets out of stimulated ones (%) | | | Duration of antennal contacts (s) | | |
| --- | --- | --- | --- | --- | --- | --- |
|  | *S.symbiotica*-free | *S.symbiotica*-infected |  | *S.symbiotica*-free | *S.symbiotica*-infected |  |
| Day 1 | 85 ± 21  N=10 | 65 ± 41  N=8 | a | 4.2 ± 1.7  N=65 | 5.0 ± 2.4  N=74 | a |
|  | PWC: p=0.9 | | | PWC: p=0.5 | | |
| Day 2 | 78 ± 34  N=9 | 84 ± 25  N=8 | a | 4.5 ± 1.7  N=76 | 5.4 ± 2.6  N=65 | a |
|  | PWC: p=0.9 | | | PWC: p=0.8 | | |
| Day 3 | 82 ± 30  N=10 | 54 ± 34  N=7 | a | 5.1 ± 3.5  N=83 | 5.4 ± 3.2  N=75 | a |
|  | PWC: p=0.9 | | | PWC: p=0.9 | | |
| Day 4 | 91 ± 9  N=10 | 35 ± 23  N=8 | a | 7.0 ± 4.6  N=74 | 5.1 ± 3.0  N=86 | b |
|  | PWC: p=0.007** | | | PWC: p=0.02* | | |
| Day 5 | 49 ± 44  N=9 | 25 ± 34  N=9 | a | 6.0 ± 2.4  N=66 | 4.1 ± 2.6  N=80 | ab |
|  | PWC: p=0.1 | | | PWC: p<0.001*** | | |
| Day 1-Day 5 | 77 ± 32  N=48 | 52 ± 37  N=40 | | 5.4 ± 3.2  N=364 | 5.0 ± 2.8  N=380 | |
|  | PWC: p<0.001*** | | |  |  |  |
| Infection effect | GLMM: p<0.001*** | | | LMM: p=0.06 | | |
| Time effect | GLMM: p=0.02* | | | LMM: p<0.02* | | |
| Interaction effect | GLMM: p=0.03* | | | LMM: p=0.001*** | | |
