# Supplementary material for: Infected connections: Unraveling the impact of a bacterial symbiont on ant-aphid partnership
Source: PLoS One. 2025 Jun 23;20(6):e0326875. doi: 10.1371/journal.pone.0326875 (PMC12184899; doi:10.1371/journal.pone.0326875)
Supplement: Attachment — Submitted filename: Responses to reviewers.docx [file pone.0326875.s012.docx]

# Responses to editor and reviewers

We would like to thank the editor as well as the two reviewers for their constructive comments and insightful suggestions. We have addressed all comments that were raised and point-by-point responses are given below in italics.

**Journal Requirements:**

When submitting your revision, we need you to address these additional requirements.

1. Please ensure that your manuscript meets PLOS ONE's style requirements, including those for file naming.

*The revised manuscript is complying with PLOS ONE's style requirements. Figures were improved and checked with PACE to match the formats required by PLOS ONE. Please note that the numbering of supplementary tables has changed (all tables about model selection are now put in successive order)*

2. In your Methods section, please provide additional information regarding the permits you obtained for the work. Please ensure you have included the full name of the authority that approved the field site access and, if no permits were required, a brief statement explaining why.

*We conducted experiments in full compliance with ethical, legal, and institutional guidelines for the collection and use of animals. According to local laws and regulations, no permits were required to collect colonies of Lasius niger ant species. Aphids originated from colonies reared in the laboratory of UCL (Université Catholique de Louvain) partner.*

*As requested, this information is now added in the revised “Materials and Methods” section on lines 379-385.*

3. Please amend either the title on the online submission form (via Edit Submission) or the title in the manuscript so that they are identical.

*Actually, the title of the online submission form was missing an "a". We will correct the title via Edit Submission to match the title of the manuscript when submitting the revised manuscript and responses to reviewers.*

4. Please review your reference list to ensure that it is complete and correct. If you have cited papers that have been retracted, please include the rationale for doing so in the manuscript text, or remove these references and replace them with relevant current references. Any changes to the reference list should be mentioned in the rebuttal letter that accompanies your revised manuscript. If you need to cite a retracted article, indicate the article’s retracted status in the References list and also include a citation and full reference for the retraction notice.

*We have corrected and completed the reference list. No reference has been removed from the revised manuscript. We have added four references to the list : three references suggested by reviewer 1 and two references. One (Detrain & Prieur, 2014) is about the impact of honeydew sugars on ant feeding and the other (Yao & Akimoto, 2001) is about the ant attendance and the honeydew composition.*

5. Review Comments to the Author

**Reviewer #1**:

**Comment 1:** A very interesting manuscript on an experiment, that sheds new light on microbiome-driven factors affecting ant-aphid mutualism. The experiment and the analyses of the results seem to be well-prepared, as well as the manuscript, although the quality of the figures could be much better for publication. I have only a few more suggestions on the manuscript:

***Response 1:*** *We would like to sincerely thank Reviewer #1 for his/her positive and stimulating comments. We have improved the quality of the figures and checked them with PACE to match the formats required by PLOS ONE.*

**C2:** Line 46, 49 – add the spaces before the brackets
Line 66 – remove the space before the comma

Line 148 – remove the space before the dot

***R2:*** *We thank Reviewer #1 for the careful review of our manuscript. All suggested changes were made.*

**C3:**Line 70 – ant-aphid mutualism also influenced aphid evolution, for details please see: Depa et al. 2020

***R3:*** *We agree with Reviewer #1 and reference to Depa et al. 2020 is now added at lines 79-82.*

**C4:**Line 135-140 – did the ant colonies contain a queen and brood? As there were nursery workers, I assume there were larvae and the demand for nutrients was constant. If not, then the ants perhaps have no occasion to utilize the energy from honeydew, and their demand for food decreased during the study.

***R4:*** *All experimental ant colonies were queenless and broodless for the following reasons. Firstly, for practical reasons, having large queen-right colonies in the laboratory requires to collect founding queens and to rear incipient colonies what may take several years to get a large number of workers. As our experiments had to be carried out on mature colonies, with a sufficiently large population of workers, we had to collect them in the field but, in this case, it is very unlikely to harvest the queen. Secondly, for standardization purposes, we wanted to limit the variability in food needs due to differences in the number of larvae or in egg-laying rate by queens.*

*Since broodless and queenless colonies were used in both treatments, we feel this does not invalidate our between-treatment comparisons and conclusion about the impact of S. symbiotica on aphid tending by ants. That being said, as rightly pointed out by Reviewer #1, ants may have a reduced need for food in the absence of brood what likely explains the decrease in honeydew consumption over time (especially on day 5). This comment is now included on lines 450-451.*

*Thanks to referee #1 comment, we also realize that using the term “nurse” for “workers staying inside the nest” was inappropriate. We have changed it to “internal workers” throughout the manuscript and specified that experimental colonies had no queens or brood at lines 249-250****.***

**C5:** Now we also know that ants do eat aphids they tend (e.g. Sakata 1994). Were there any cases of preying on attended aphids, which might have influenced the rate of the aphid colony growth?

***R5:*** *Indeed, given that ants may occasionally prey on aphids, we looked for any Serratia-driven shift from tending to predation. Therefore, we looked for any aggressive behaviors exhibited by the ants, including predation on aphids, bites and threatening behavior. However, over the whole duration of video recordings (4500 minutes in total),* *we never observed any agonistic behaviors or predation by ants towards S. symbiotica-infected or uninfected aphids. Furthermore, in the case of low aphid densities, as in our experiment where the highest number of aphids ranged between 131 and 200 aphids, Sakata et al. (1995) scarcely observed predation on aphids which were primarily used by the ants as honeydew producers.*

*We have added a sentence about ant predation on aphids as well as related references in the introduction (see lines* *86-88). We also mention in the revised “Materials and Methods” section that we paid attention to agonistic behaviors (lines 289-291) and in the result section that none was observed (lines 542-544).*

**C6:** With the M&M section, I realise that, as you stated, certain interrelations between ant-ant aphids ceased to occur after Day 5, but providing these further results somehow would highlight the meaning of these relations while they occurred, just by setting the broader background.

***R6:*** *We are not sure to have well-understood this comment.* *If reviewer #1’s comment is linked to the observed decrease (not cessation) of aphid attendance by ants after day 5, we agree that it may be surprising since several studies showed an increase in the number of ants visiting plants with increasing numbers of aphids and honeydew production (Way, 1963; Völkl et al., 1999; Stadler & Dixon, 1998; Katayama & Suzuki, 2003). It is nevertheless worth to notice that a decline over time in the number of ants on plants infested by aphids has already been observed in previous studies by Breton and Addicott (1992). As rightly pointed out by Reviewer #1 (see C4), we think that, in our study, the decrease in the ants' interest in aphids is likely due to the absence of brood, and hence limited demand for energy.*

*If reviewer #1’s comment is rather questioning possible changes in the nature of ant-aphid interactions over time (after day 5), we found no discernible change in ant-aphid relationships: while they became less frequent, interactions did not change qualitatively, in particular, there was no shift from nutritional interaction to predation (see also R5).*

*As for providing further results, interaction indices after Day 5 were not included in the manuscript because contacts between ants and aphids became too few to carry out any relevant statistical analysis.*

**C7:** Line 506-509 – I have a feeling that it might be safer to use the term “modify” than “weaken”. A. fabae is facultatively myrmecophilous, which means there are cases when they are aggressively defended by ants, and there are cases when they are not ant-attended at all, and that does not mean their colonies are in bad condition. In your case, indeed, Serratia weakened the mutualistic engagement of ants, but it is too weak a result to generalize it to the statement that it may weaken the ant-aphid mutualism. Unless, of course, we prove that many more facultatively myrmecophilous aphid species do have Serratia when unattended and no Serratia (or some other bacteria) when ant-attended.

***R7:*** *We agree with Reviewer #1 that we lacked caution by generalizing the weakening effect of Serratia symbiotica on Lasius niger-Aphis fabae interaction. As suggested, we have replaced the term “weaken” by “modify” at line* *594****.***

**C8:**For the purpose of the Discussion, it might be beneficial to refer to Kaszyca-Taszakowska & Depa 2022 paper, where also aphids unattended by ants have a higher proportion of Serratia in their facultative microbiome. The results of the submitted manuscript may largely explain that report, via different explanations of the influence of Serratia on ant-aphid mutualism. Additionally, I would suggest referring to the paper by Ivens et al. 2018, where Serratia issues may also be interconnected with the data in the manuscript.

***R8:*** *We thank Reviewer #1 for this comment. The suggested references have actually demonstrated that a variable effect of social partnership in shaping a host symbiotic microbiome. It namely depends on whether the microbiome is vertically transmitted between generations, with a predominant phylogenetic component (as shown in aphid trophobionts by Ivens et al. 2018), or whether it is a more open association likely to be subject to horizontal and social transmission (as for Acetobacteraceae bacteria) in aphid-tending ants (Ivens et al. 2018). In turn, our results suggest that the host microbiome, specifically the presence of S. symbiotica, can alter social partnerships such as the trophobiotic interactions between ants and aphids. The disinterest in the honeydew produced by S. symbiotica-infected aphids is likely to lead to a decrease in ant attendance. We fully agree with Reviewer #1 that this can, at least partially, explain why aphids that were not attended by ants showed a higher proportion of Serratia in their microbiome (Kaszyca-Taszakowska & Depa 2022 but see Henry et al. 2015). This interesting suggestion as well the two references have been added to the discussion on lines 596-604* *and lines* *719-723.*

**Reviewer #2**: After numerous studies on the effects of endosymbiotic bacteria on aphid ecology, your research provides valuable insight into the effects of gut-colonizing bacteria on aphid populations and their interactions with aphid-tending ants. I found this well-designed study to be highly interesting, and I am intrigued by the idea that the boundaries between plant-benefitting bacteria and aphid symbionts can be so blurred. However, I have identified several points that need to be addressed prior to publication.

***R9:*** *We thank Reviewer #2 for these positive and encouraging feedbacks. We have addressed all your comments in order to improve the revised manuscript.*

Introduction:

**C10:**Your introduction is compelling and requires only minor corrections (see comments below). Please double-check your punctuation, as there are numerous comma errors that I did not highlight individually.

**R10:** *Thanks. We have carefully reviewed and corrected all the typographical and grammatical issues, including punctuation inconsistencies.*

Methods:
**C11:**You have described the procedure for infecting aphids with the bacterial strain in detail, but it is unclear whether you verified the success of this procedure. Typically, a diagnostic PCR is performed to confirm infection status before proceeding with experiments involving endosymbiotic bacteria. Do you have data demonstrating that your infection procedure is sufficiently reliable to justify not testing infection status beforehand?

**R11:** *We acknowledge that the submitted manuscript lacked information on how we checked the infection status of aphids. We performed diagnostic PCR on 5 aphids collected from the patch of uninfected and on 5 aphids from S. symbiotica-infected patch, both patches being further used for the experiments. This was conducted five days post-infection, corresponding to the time at which aphids were placed onto the experimental plants. Additionally, the infection status of the aphids was reassessed 20 days after being fed on the artificial diet. These diagnostic PCR were carried out on 40 aphids: 10 individuals collected for each of the 4 tested experimental conditions: 1) S. symbiotica-infected aphid with tending ants, 2) uninfected aphids with tending ants, 3) S. symbiotica-infected aphid without ants and 4) uninfected aphid without ants. The details of PCR procedure are provided in the manuscript. PCR products were visualized on 1% agarose gels.*

*We added images of gels in our data repository (https://doi.org/10.5281/zenodo.15342394). As shown in the first gel image, the first five wells containing the PCR products of uninfected aphids showed no visible bands. In the five following wells containing the PCR products of aphids infected by S. symbiotica for 5 days, bands corresponding to S. symbiotica (500 bp) were visible. This confirms the infection status of aphid patches used in the experiments. On the second and third gel images**, each set of four wells corresponded to one ant colony: the first two wells contained the PCR products of uninfected aphids (attended and unattended by ants), and the following two wells contained the PCR products of S. symbiotica-infected aphids (attended and unattended by ants). In these last wells, bands corresponding to S. symbiotica were clearly visible, confirming the infection status.*

*Finally, diagnostic PCR were also performed on frozen stocks of CWBI-2.3^T^ cultivable strain that was used for the oral infection, as well as on the infected artificial diet. Those PCR products were sequenced by Macrogen (Netherlands) to ensure the strain of bacteria given to the aphids.*

*This information is now addressed and detailed in the “Materials and Methods” section (see lines 198-219).*

**C12:**I also found your aphid counting methods somewhat unclear. Why did you count only adults and third instar nymphs, despite A. fabae having four nymphal stages? This methodology may explain why population growth only began on day 5, when the first nymphs reached late instars. If there is a valid reason for omitting early instar aphids, please mention it and clarify the rationale for this approach.

***R12:*** *Actually, although Aphis fabae aphids have four nymphal stages, we counted the number of aphids each day starting from the 3^rd^ nymphal instar onwards for the following practical reasons. First, early nymphal stages are very small, making by-eye counting difficult and error-prone. Furthermore, discriminating 1^st^ and 2^nd^ nymphal instars can be particularly challenging due to many morphological similarities. Finally, it was impossible to dismantle the setup to remove the aphids, count all stages, including the early ones, and then reintroduce them onto the plant without deeply disturbing the experimental procedure. To ensure consistency in data collection, the same observer conducted all daily counts starting from the 3rd nymphal instar.*

*We agree that this may partially explain that aphid population growth started only on the fifth day of the experiment, which coincides with the emergence of the late nymphal instars. This information has been added in lines 237-243 (materials and methods) and lines 399-401 (results).*

Results:
**C13:** Would it be possible to merge certain figures (e.g., Figures 3, 4, and 5) and move relevant supplementary data into the main text? Doing so could improve readability and reduce the need for frequent cross-referencing. Additionally, consider refining the layout of figures to enhance clarity (see detailed comments below).

***R13:*** *We thank Reviewer 2 for this suggestion. The former figure 3 and 4 are now merged and the S5 Table, about number of honeydew droplets production, has been incorporated in the main text of the revised manuscript, as Table 1. Figures have been improved and checked with PACE to match the formats required by PLOS ONE. Supplementary files and figures have been re-numbered.*

**C14:**Use consistent terminology throughout the results section (e.g., always use “infection status”), and present results in a logical order, such as addressing main effects before interaction effects (see specific comments below).

***R14:*** *We have removed inconsistencies in terminology and presented the main effects before the interaction ones (see lines 490-493).*

Discussion:
Your discussion is engaging and generally well-written. However, there are a few points that I recommend addressing or expanding upon:

**C15:** Consider discussing the effect of ants on aphid honeydew quality and excretion (see comments on line 560). Previous studies suggest that ant-tending increases both the quality and quantity of honeydew. It is worth considering whether S. symbiotica-infected aphids were unable to improve honeydew quality over time, leading to reduced ant attention.

**R15:** *Actually, we suspect that some alteration of the physiological condition of S. symbiotica-infected aphids may have prevented them to improve their honeydew quality over time, resulting in a reduced attention from ants. We agree with Reviewer #2 that honeydew analysis would be an interesting line of research. We have introduced this idea in the discussion section on lines 658-661.*

**C16.** Highlight the potential confounding effect of Serratia infection in both aphids and plants on ant behavior and plant occupation (see comments on lines 602–608). Given that ants might be collecting nectar from EFNs instead of honeydew from aphids, you should either acknowledge this limitation or explain why your plant occupancy index remains valid.

***R16:*** *This is actually an important issue worth to be discussed. To our knowledge, there are no paper investigating the effects of S. symbiotica on extrafloral nectaries in Serratia-infected plants. The extent to which a bacterium influences the production and quality of nectar in EFNs is certainly a future research to be carried out.*

*Regarding the plant occupancy index, we believe that extrafloral nectaries have only a very marginal effect on the number of foragers mobilized towards the plants. Indeed, in terms of nutrient quantities, the sugary production of extrafloral nectaries remains negligible when compared to the aphids. Vicia faba shows a pair of extrafloral nectaries on the underside of the stipules (Kirk, 2004. Bee World, 85(03), 60-62. DOI: 10.1080/0005772X.2004.11099625); Katayama and Suzuki, 2003). Since the plants were at the physiological stage of BBCH12 at the beginning of the experiment, they possessed 4 active EFNs. At the end of the experiment, plants were usually at stage BBCH14 with a maximum of 8 EFNs, thus producing an estimated amount of 0.05-0.10µl of extrafloral nectar (Mondor et al,. 2013. Open Journal of Ecology, 03(01), 59-65. DOI: 10.4236/oje.2013.31008). Thus, we think that the plant occupancy index and the number of mobilized foragers were mainly influenced by the production of honeydew rather than that of EFNs. This does exclude a further impact of extrafloral nectaries on the spatial distribution of ant foragers over the plant and hence a contribution to its defense against herbivory. This information was added on lines 701-704.*

**C17.**Finally, please review the punctuation in the discussion section, as I noticed several comma errors that I did not mark individually.

***R17:*** *Done. Thanks for noticing these punctuation inconsistencies.*

**C18.** Specific comments and suggestions:
Line 34: …large amounts (plural)
Line 51: remove the “a” before better
Line 63: The comma needs to be before the “but” and not after
Line 90: …exhibit variable degrees of… (no “a” and degrees in plural)
Line 103: I would suggest rewriting the middle part of this sentence: …(29, 54-56) , allowing manipulation of its occurrence in…
Lines 135-136: …aphid-tending species that is widespread in temperate regions of Europe.

***R18:*** *All the suggested corrections have been made. Thanks.*

**C19** Line 145: To my knowledge Christoph Vorburger is a Professor at ETH Zürich and not at the University of Zürich.

***R19:*** *Reviewer #2 is absolutely right, it has been corrected on line 161****.***

**C20:** Line 168: Finally with double l.
Line 171: …protocols found in the literature.
Line 180: …except that sterile PBS … (not excepting)
Lines 207-208: …before the beginning of the experiment… (the missing)
Line 240: The recorded images covered an area… (no “on” needed)
Line 252: , we measured the time elapsed between… (time is not a plural)
Line 281: We included the infection status… (not infectious)
Line 296: infection status (not infected status)

**R20:** *All the suggested corrections have been made in the revised manuscript.*

**C21** Line 329: Always use same terminology to improve clarity. S. symbiotica-free and S. symbiotica-infected

**R21**: *The same terminology is now used throughout the revised manuscript.*

**C22:**Lines 337-339: Move this sentence to the methods section.
Lines 377-379: Move this sentence to the methods section.

**R22:** *These two sentences have been incorporated into the statistical analyses part of the “Materials and Methods” section, on lines 327-328 and lines 342-343. We have also added a sentences on model selection for the part on “aphid-tending behavior and honeydew consumption by ants” to maintain consistency of the statistical analyses section, see lines 370-371.*

**C23**Lines 416-417: Unclear sentence. Suggestion: Indices significantly lower than the theoretically expected value of 50%.

**R23:** *Done. Thanks for the suggestion.*

**C24**Lines 421-426: Mention the main effect before the interaction effect. Is “infection status” the same as “infection condition”? Please use consistent terminology throughout the manuscript.

**R24:** *The term “infection status” and “infection condition” meant the same. To avoid confusion, we kept only the “infection status” terminology. The fixed effects is now mentioned before the interaction effect (see R14).*

**C25:**Line 452: …according to the best… (to missing)
Line 453: We however found a significant… ("a" is missing)
Lines 478-479: I think there is a word missing in this sentence. (…aphids would be(?) twice that of…)

**R25:** *Thanks.* *All the corrections have been done.*

**C26:**Line 560: Maybe mention that the presence of ants can also change the composition of released honeydew (shown in your reference 76). Maybe S. symbiotica infected aphids were not able to increase honeydew quality over time and thus received less ant attention.

Lines 602-608: According to what you say here, ant behaviour can be influenced by the effect of the bacteria on the aphid or on the plant since plants can also get infected with this Serratia strain. This confounding effect impacts your plant occupancy index because ants observed on a plant might be collecting nectar from EFNs rather than honeydew from aphids. Please mention this restriction and/or clarify why your plant occupancy index is still useful.

***R26:*** *please refer to our previous response (R15 and R16) to these comments.*

**C27**Table S1: replace “Colonial ID” with “Colony ID”

***R27:*** *Done.*

**C28:** Figures: If there is space, I would find it clearer to add a legend to the plots than describing the meaning of the colours in each caption.

***R28:*** *We have added a color legend in Figure 3 but for the rest of the figures, not to add a color legend to the plots to avoid cluttering them. The meaning of colors is still detailed in the caption*

**C29:** Figure 4: You mention in the caption that “Significant differences are shown for pairwise comparisons…” but I cannot see any bars or lines indicating differences in the Figure.

**R2*9:*** *We thank Reviewer #2 for this comment. Since the former figures 3 and 4 are now merged, the caption has been fully reedited (see R13).*

**C30** Figure 5: Why does this figure have horizontal lines while none of the others does? Please adapt.

**R30:** *Done. Horizontal lines were useless and thus removed.*
